# Omega 3 fatty acids induce a marked reduction of apolipoprotein B48 when added to fluvastatin in patients with type 2 diabetes and mixed hyperlipidemia: a preliminary report

**DOI:** 10.1186/1475-2840-8-1

**Published:** 2009-01-08

**Authors:** Pedro Valdivielso, José Rioja, Carlota García-Arias, Miguel Angel Sánchez-Chaparro, Pedro González-Santos

**Affiliations:** 1 From Lipids Unit, Department of Medicine, Hospital Virgen de la Victoria, Malaga and Department of Medicine & Dermatology, University of Malaga, Malaga, Spain; 2 Centro de Investigaciones Médico-Sanitarias, Department of Medicine & Dermatology, University of Malaga, Malaga, Spain

## Abstract

**Backgorund:**

Mixed hyperlipidemia is common in patients with diabetes. Statins, the choice drugs, are effective at reducing lipoproteins that contain apolipoprotein B100, but they fail to exert good control over intestinal lipoproteins, which have an atherogenic potential. We describe the effect of prescription omega 3 fatty acids on the intestinal lipoproteins in patients with type 2 diabetes who were already receiving fluvastatin 80 mg per day.

**Methods:**

Patients with type 2 diabetes and mixed hyperlipidemia were recruited. Fasting lipid profile was taken when patients were treated with diet, diet plus 80 mg of fluvastatin and diet plus fluvastatin 80 mg and 4 g of prescription omega 3 fatty acids. The intestinal lipoproteins were quantified by the fasting concentration of apolipoprotein B48 using a commercial ELISA.

**Results:**

The addition of 4 g of prescription omega 3 was followed by significant reductions in the levels of triglycerides, VLDL triglycerides and the triglyceride/HDL cholesterol ratio, and an increase in HDL cholesterol (P < 0.05). Fluvastatin induced a reduction of 26% in B100 (P < 0.05) and 14% in B48 (NS). However, the addition of omega 3 fatty acids enhanced this reduction to 32% in B100 (NS) and up to 36% in B48 (P < 0.05).

**Conclusion:**

Our preliminary findings therefore suggest an additional benefit on postprandial atherogenic particles when omega 3 fatty acids are added to standard treatment with fluvastatin.

## Background

Type 2 diabetes is characterized by hypertriglyceridemia, low concentrations of HDL cholesterol, increased small-dense LDL (sdLDL), greater postprandial lipidemia and a considerable increase in vascular risk [1]. It has been clearly established that LDL cholesterol levels should be kept below 100 mg/dL (with an optional goal of < 70 mg/dL) or, if hypertriglyceridemia is present, a non-HDL cholesterol < 130 mg/dL (optional < 100 mg/dL). Together with dietary recommendations and lifestyle changes, statins are the drugs of choice for this goal [2].

Postprandial lipoprotein particles have been shown to be atherogenic in experimental animal [3], case-control [4] and histological studies [5]. Indeed, nonfasting triglycerides predict the vascular risk better than fasting measurements [6]. Additionally, postprandial triglycerides are independently associated with carotid [7] and peripheral atheromatosis [8] in patients with type 2 diabetes. Accordingly, it seems reasonable to seek additional control of the triglycerides in patients with mixed hyperlipidemia. We therefore undertook a preliminary evaluation of the effect of the addition of omega 3 fatty acids on the postprandial particles, measured specifically as plasma concentrations of apolipoprotein B48, in diabetic patients with mixed hyperlipidemia receiving treatment with fluvastatin.

## Patients and methods

We undertook a non-controlled, open-label study of the lipid effects of omega 3 fatty acids when they were added to a low-calorie diet and fluvastatin in patients with type 2 diabetes and mixed hyperlipidemia. The diabetic patients, who had an acceptable metabolic control, were selected from those who attended the Lipids Unit with mixed hyperlipidemia. Patients were excluded if they had known vascular disease or if they refused to participate. The study was approved by the Ethics and Research Committee of the Virgen de la Victoria Hospital, Malaga.

The patients were seen on four occasions. The first visit was to select and obtain the informed consent of the patients. At this visit the patients were instructed to cease taking any lipid-lowering treatment and instructed to follow a low-calorie diet for six weeks. This diet was 600 kcal below the basic calorie needs, as calculated by the Harris and Benedict equation [9]. At the second visit, six weeks after starting the low-calorie diet, a 12-hour fasting blood sample was obtained and the patients were requested to continue the diet advised and to take 1 prolonged-release 80 mg fluvastatin tablet at night until the end of the study. At the third visit, eight weeks after starting the treatment with fluvastatin 80 mg, a further blood sample was taken and, in addition to the diet and the fluvastatin 80 mg, the patients were asked to take 4 capsules of prescription omega 3 fatty acids daily, containing 460 mg of EPA and 380 mg of DHA ethyl esters. The patients were examined 8 weeks later at the final visit. The clinical variables assessed at each visit were weight, waist circumference, blood pressure, degree of treatment compliance, adverse effects and a subjective evaluation of the dietary compliance.

### Laboratory variables

After a 12-h overnight fast, a sample of blood was drawn to measure levels of glycemia, glycated hemoglobin, insulin, lipids and lipid fractions. Serum glucose was measured with a Cobas Integra (Roche) autoanalyzer, using a hexokinase-based enzymatic method. The glycated hemoglobin was measured by HPLC (ADAMS-A1c HA 8160). The plasma lipids, cholesterol and triglycerides were measured with enzymatic techniques (HORIBA-ABX, Montpellier, France) in a Cobas Mira autoanalyzer. Lipoproteins were separated by β quantitation (ultracentrifugation) using the Lipid Research Clinics Protocol. The levels of apolipoprotein B48 were quantified with a high-sensitivity commercial ELISA (Shibayagi Co Ltd, Ishihara, Japan). Plasma levels of apolipoproteins were measured by immunoturbidimetry; A1 and B100 from Incstar, Diasorin, Stillwater, OK, USA and C-II and C-III from Daichii Pure Chemicals, Kyoto, Japan.

### Statistical studies

The variables are shown as the mean ± SD. To compare the changes in the different variables over the three study points we used the Student *t *test for paired data, because the normality assumption was confirmed for all the variables. The analysis was made with SPSS 15.0 (SPSS Inc, Chicago, IL)

## Results

The study initially included nine patients, but one withdrew voluntarily at the start; the results therefore refer to eight patients, five men and three women, aged 57 ± 5 years. Six patients were hypertensive and all were receiving treatment with an angiotensin-converting enzyme (ACE) inhibitor or an angiotensin receptor antagonist II (ARA-II). No patient had experienced any cardiovascular event. Three patients were treating their diabetes with diet, another three with metformin, one with glycazide and another with glycazide plus metformin. The low-calorie diet, blood-pressure lowering treatment and antidiabetic medication remained unchanged throughout the study period.

**Figure 1:**
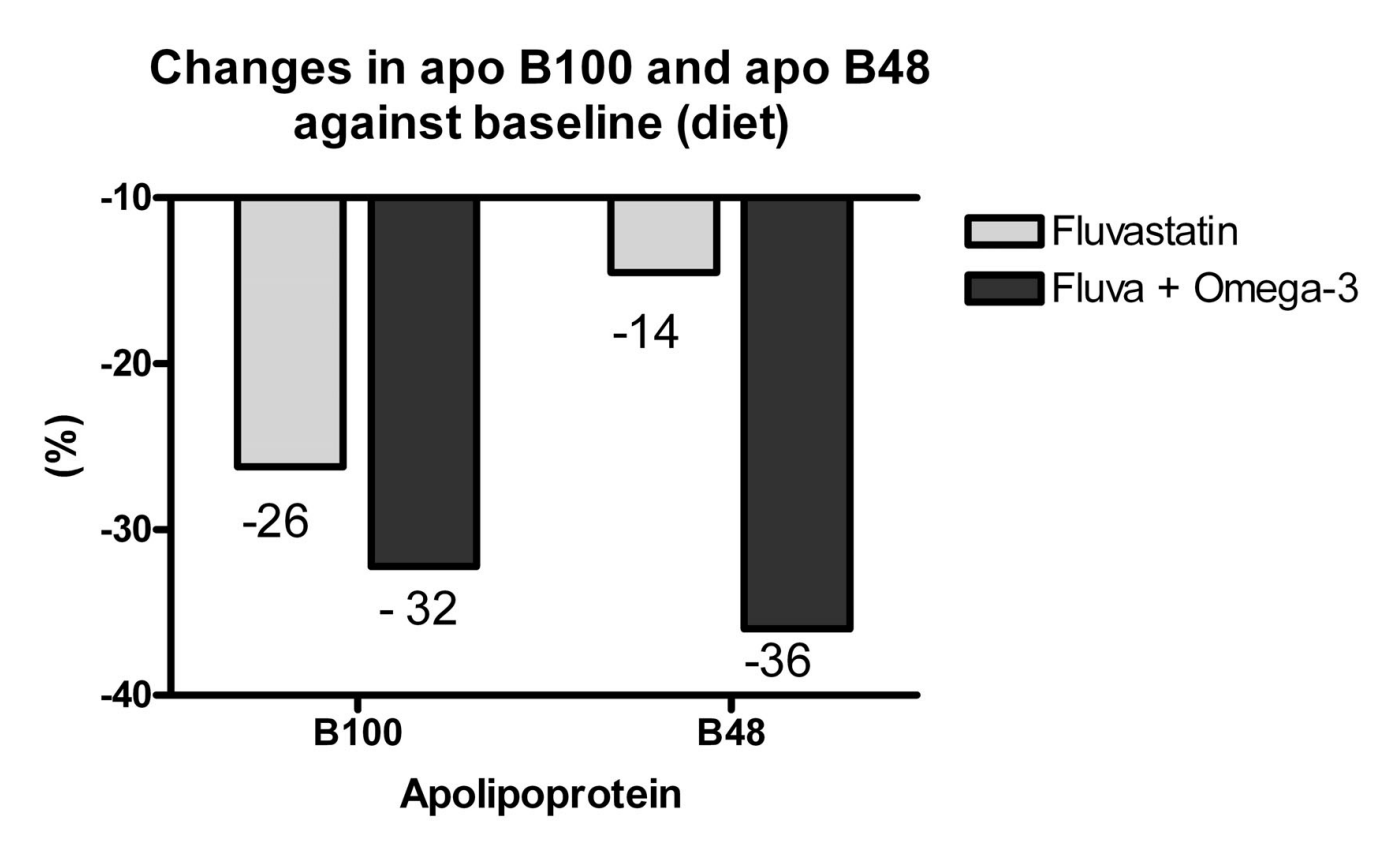
**Plasma levels of apolipoprotein B100 and apolipoprotein B48 according to treatment period**. The reduction in apolipoprotein B100 was significant after adding fluvastatin 80 mg to the diet, whereas apolipoprotein B48 only fell significantly after the addition of 4 g of omega 3 fatty acids to the diet and the fluvastatin 80 mg.

Table 1 shows the clinical characteristics of the study patients. All were overweight or obese and had adequate glycemic control during the study period. Table 2 shows the significant reduction in the levels of total cholesterol, LDL cholesterol, apolipoprotein B100 and apolipoprotein C-III, and an increase in the C-II/C-III ratio two months after starting treatment with fluvastatin. The addition of 4 g of prescription omega 3 fatty acids was followed by a significant reduction in serum triglycerides, VLDL triglycerides and the triglyceride/HDL cholesterol ratio and a significant increase in HDL cholesterol. Apolipoprotein B48, the main outcome variable in this study, only fell significantly after the addition of 4 g of omega 3 fatty acids to the statin treatment (Fig 1).

**Table 1:** Anthropometrical data, blood pressure and glycemic parameters.

	(A) Diet	(B) Diet plus Fluvastatin 80 mg	(C) Diet plus Fluvastatin 80 mg plus Omacor 4 g
Weight (Kg)	79 ± 14	78 ± 15	78 ± 14

BMI (kg/mt^2^)	31.3 ± 3.5	30.8 ± 4.0	30.7 ± 3.8

Waist Circunference (cm)	106 ± 8	105 ± 10	105 ± 10

Glucose (mmol/L)	10.2 ± 3.2	10.8 ± 3.4	11.2 ± 2.8

HbA1_c _(%)	6.56 ± 0.75	6.56 ± 1.10	6.61 ± 1.16

Insulin (μU/ml)	15.4 ± 11.7	13.5 ± 8.0	14.2 ± 10.9

HOMA-IR	7.6 ± 7.4	7.1 ± 6.5	8.0 ± 8.3

All changes did not reach statistical significance

**Table 2:** Fasting lipids, lipoproteins and apolipoproteins in the three periods of treatment

	(A) Diet	(B) Diet plus Fluvastatin 80 mg	(C) Diet plus Fluvastatin 80 mg plus Omacor 4 g
Total Cholesterol^ac^	259 ± 55	174 ± 35	203 ± 54

LDL cholesterol^ac^	157 ± 47	103 ± 25	127 ± 30

HDL cholesterol^bc^	34 ± 9	32 ± 10	40 ± 11

Non-HDL chol^ac^	225 ± 55	142 ± 35	163 ± 54

Triglycerides^c^	337 ± 153	263 ± 226	195 ± 129

VLDL triglycerides^c^	289 ± 160	224 ± 222	136 ± 118

Tg/HDL-chol ratio^c^	4.80 ± 2.69	4.39 ± 5.10	2.23 ± 1.56

Apo A-1	113 ± 24	107 ± 41	124 ± 26

Apo B100^ac^	126 ± 34	89 ± 16	82 ± 16

Apo B48^c^	5.81 ± 2.59	4.12 ± 2.78	3.07 ± 1.47

Apo C-II^ac^	20.4 ± 6.1	18.1 ± 5.6	15.8 ± 6.8

Apo C-III ^a^	18.7 ± 5.7	12.9 ± 3.5	12.8 ± 4.7

C-II/C-III ratio	1.13 ± 0.31	1.41 ± 0.29	1.30 ± 0.16

Data showed as mg/dL except apo B48, in mg/L.

^a ^(A) vs (B) p < 0.05.

^b ^(B) vs (C) p < 0.05.

^c ^(A) vs (C) p < 0.05.

## Discussion

Omega 3 fatty acids have long been used in the treatment of hypertriglyceridemia, as they reduce the hepatic production of VLDL, favor fatty acid oxidation and enhance VLDL clearance [10]. They have also been shown to reduce levels of chylomicrons and their remnants, as well as the amount of apolipoprotein B48 [11]. The reduction in the concentrations of triglycerides and VLDL triglycerides and the increase in HDL cholesterol noted in this study are thus in agreement with the literature mentioned. Concerning LDL cholesterol, treatment with omega 3 fatty acids in patients with hypertriglyceridemia or mixed hyperlipidemia is usually followed by a slight rise in LDL cholesterol [12]. Although in our study, LDL cholesterol rose slightly, but not significantly after the omega 3 treatment, the marked reduction in the triglyceride/HDL cholesterol ratio noted suggests the transformation of sdLDL to larger particles [13].

Evidence exists for the beneficial effect on non-HDL cholesterol of the addition of omega 3 fatty acids to the treatment in persons with residual hypertriglyceridemia despite the use of statins [14]. However, their benefit on the intestinal atherogenic lipoprotein particles has not been assessed.

The quantification of apolipoprotein B48 by ELISA is a simple way to measure postprandial metabolism, without the need for ultracentrifugation, sample delipidation and gradient electrophoresis, a very costly process. Apolipoprotein B48 is specific for the intestinal lipoproteins [15] and its fasting levels correlate very well with the area under the curve after a fatty meal [16]. Its levels are raised in persons with type 2 diabetes, especially when they also have renal failure [17].

The effects of fluvastatin on the concentration of apolipoprotein B48 has not been reported. Nevertheless, atorvastatin is able to reduce apolipoprotein B48 concentrations by increasing the catabolism of chylomicrons and their remnants [18,19]. This study supports the hypothesis that levels of B48-containing atherogenic lipoproteins may be further reduced by omega 3 fatty acids, even when added to the traditional statin therapy; in fact, the reduction in the B48 was over two-fold greater with the omega 3 fatty acids than with 80 mg of fluvastatin alone (Figure 1). Because we just evaluated the patients over eight weeks, the reduction in the levels of apolipoprotein B48 might be even more pronounced over time.

The apolipoprotein B48 concentrations only fell significantly after the incorporation of omega 3 fatty acids to the treatment with fluvastatin. As treatment with omega 3 fatty acids in patients with hypercholesterolemia who are receiving statins is accompanied by a reduction in ischemic events and vascular death, even in populations that have a high consumption of fish [20], we may speculate that the reduction in apolipoprotein B48-containing particles might contribute to this benefit. It is believed that omega 3 fatty acids enhances chylomicron clearence due to a reduction in hepatic VLDL synthesis [11]. Moreover, in experimental models omega 3 fatty acids may also reduce intestinal lipoprotein production [21], which is increased in patients with insulin-resistance [22] and type 2 diabetes [23].

In conclusion, these preliminary findings suggest that treatment with 4 g of omega 3 fatty acids added to higher doses of fluvastatin in diabetic patients with mixed hyperlipidemia is accompanied by a significant additional benefit in apolipoprotein B48-containing particles and may thus represent a complementary therapy for the reduction of LDL cholesterol, non-HDL cholesterol and B100 to that achieved with statins alone. Nevertheless, due to small sample size, lack of postprandial data and open-label design, large-scale, double-blind, randomized studies are needed to confirm our results.

## Abbreviations

sdLDL: small-dense LDL.

## Competing interests

PVF reports receiving consulting and lecture fees from Ferrer-Novag, the company who financed the study.

## Authors' contributions

PVF designed the study, reviewed statistical analysis and wrote the draft. JR performed all analytical and statistical procedures. CG and MASCH recruited and looked after patients and reviewed the draft PGS participated in its design and coordination and helped to draft the manuscript. All authors have also made substantive intellectual contribution to the study and have approved the final manuscript.
